# Association between cardiorrespiratory fitness and cognitive control: is somatic maturity an important mediator?

**DOI:** 10.1186/s12887-022-03777-2

**Published:** 2022-12-06

**Authors:** Vinícius Muller Reis Weber, Julio Cesar da Costa, Leonardo Alex Volpato, Marcelo Romanzini, Jose Castro-Piñero, Enio Ricardo Vaz Ronque

**Affiliations:** 1 Laboratory of Physical Activity and Health, Center of Physical Education and Sports, Londrina State University - UEL, University Campus, Highway Celso Garcia Cid, Km 380, P.O. box 6001, Londrina, Paraná 86051-990 Brazil; 2 Department of Physical Education, GALENO Research Group, School of Education, University of Cadiz, Puerto Real, Spain; 3 Biomedical Research and Innovation Institute of Cadiz (INiBICA) Research Unit, 11009 Cadiz, Spain

**Keywords:** Cognition, Development, Executive Function, Working Memory, Physical fitness, Maturity

## Abstract

**Background:**

Recently some articles presented information related to the possible effect of maturity over the cognitive control and cardiorespiratory fitness, however little is known about the real effects of maturity in the relation of these variables. In this sense, the purpose of this study was to examine the potential mediating role of somatic maturity on the association between cardiorespiratory fitness (CRF) and cognitive control.

**Methods:**

This three-year longitudinal research comprises two data collection groups: a baseline conducted in 2016 with 394 adolescents (aged 11.7 ± 0.6 years) and a follow-up in 2019 with 134 adolescents (aged 14.9 ± 0.7 years). Anthropometry data, 20-m shuttle run test and peak height velocity (PHV) to determine the maximum oxygen uptake (VO_2max_) and somatic maturity, respectively, were collected at both sampling times. In parallel, the Sociodemographic and cognitive control function variables were included in the follow-up to evaluate the inhibitory control (by the Stroop test) and the visuo-spatial working memory (by the Corsi block-tapping test). Associations between CRF and cognitive functions were computed by multiple linear regression, with mediation as a function of PHV.

**Results:**

CRF exhibited transversal associations with reaction time in congruent (β = -0.004; *p* = 0.001) and incongruent (β = -0.005; *p* = 0.004) stimulus-responses. Meanwhile, the variation in VO_2max_ over the three year-study had a significant impact on the reaction time of congruent (β = -0.006; *p* = 0.001) and incongruent (β = -0.006; *p* = 0.012) responses at follow-up. However, PHV did not show a significant association with the cognitive functions, indicating no mediating role.

**Conclusions:**

Although the associations between CRF and the cognitive functions exhibited great transversal and longitudinal impacts, somatic maturity did not affect the cognitive control functions, associating exclusively with CRF.

**Supplementary Information:**

The online version contains supplementary material available at 10.1186/s12887-022-03777-2.

## Introduction

Biological maturity is the process that occurs in all body tissues, systems and organs and indicates progress towards the mature state, and the term maturity refers to the phase in which specific events related to chronological time occur (i.e., dental, somatic, sexual, and skeletal maturity) in progress to mature state [1]. The age at peak height velocity (APHV) is the most commonly used somatic maturity indicator, and PHV refers to the maximum growth rate during adolescence, at which there is significant increase in the release of sex and growth hormones, giving rise to substantial musculoskeletal development [2].

Somatic maturity influences cardiorespiratory fitness (CRF), since individuals classified in the right time exhibit higher CRF compared to late ones [3]. The positive interactions between biological maturity indicators and CRF suggest a direct interaction between maturity and CRF [4], which indicates an impact of biological maturity on CRF.

In terms of cognitive performance, CRF positively affects working memory [5, 6], inhibitory control, reaction time [5, 7, 8] and brain network [9]. Previous studies have reported that the increase in CRF had a shared variance of 53% over the increase in accuracy over time when evaluating longitudinal relationships. In cross-sectional analyses, CRF accounted for approximately 13% of reaction time and 10% of accuracy in the Flanker test [5]. Moreover, cognitive control is associated with academic achievement of adolescents, being essential to achieve good performance in school disciplines, mainly those related to math and spelling [10].

When assessing body maturation processes and performance of cognitive functions, cognitive control is strongly dependent on brain maturation, especially in the prefrontal cortex [11]. Even though the brain reaches 95% of its size by the age of six years, its development is not yet complete. The increase in neurogenesis, brain connectivity, dendritic growth and other brain processes reach their peak during adolescence, and some can persist throughout life [12–14]. However, little is known about the impact of maturation of other body systems on cognitive control. Goldstein [15] reported some effects of bone maturity on cognitive ability, possibly attributable to changes in physical and endocrine development. The release of sex hormones, especially testosterone, which is involved in the activation of synaptogenesis-specific proteins, can further activate the prefrontal cortex [14, 16]. In addition, somatic maturity is used to assess changes in physical development. Individuals who have reached PHV exhibit greater motor skills and higher hormone levels [2], which can reflect on cognitive control.

Many studies use either sexual maturity [7, 17–19] or somatic maturity (i.e., PHV) [20] as confounding variable to control their analysis, once maturity could have impacts on cardiorespiratory fitness and cognition. Control performed by confounding variables, which do not have impact on dependent and independent variables, can reduce the analysis capacity [21]. In this sense, the possible confusing role of somatic maturity in the relationship between CRF and cognitive control still requires elucidation. Therefore, the present study aims to identify the potential mediation effect of somatic maturity on the association between CRF and cognitive control. The present study hypothesizes that somatic maturity has mediation effects on the relationship between cardiorespiratory fitness and cognitive control variables.

## Methods

### Sampling and experimental design

The baseline comprised data from 394 adolescents aged 11.7 ± 0.6 years, of which 52% were female, from elementary school. Regarding the sampling process in the baseline, all the public schools of Londrina – Brazil were divided into five regions (north, south, east, west, and centre) and two schools were randomly selected from each region. Thus, classes from elementary school were randomly selected from the selected schools and all the students of the selected classes were invited to participate in the study. From 690 initial participants, only 394 presented complete data.

The follow-up conducted three years later, included data from 155 individuals aged 14.9 ± 0.7 years, of which 53% were female, from high schools of Londrina. The participants’ parents were given an additional questionnaire on learning difficulties. The final sampling excluded the students who were identified as having a learning disability (*n* = 134).

Sample size calculation for multiple linear regression analyses were conducted with the G*power software, with a 90% power, 5% error, 0.15 effect size and five predictors. Sample size calculation resulted in 116 individuals. The effect size was set at 0.15, as no previous study with this characteristic in adolescent was found; and it is a conservative value that can avoid the error cause by small sample sizes [22].

All assessments were performed individually without the influence of other participants. Cognitive tests were carried out in a quiet, noise-free environment.

**Fig. 1:**
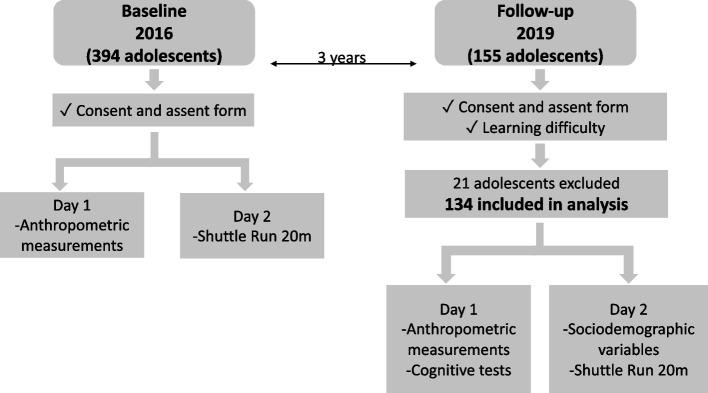
Experimental design

The experimental design is present in Fig. 1. All information took around 4 h to be collected on follow-up and 2 h on the baseline in each school. Each school had, on average, 20 students. All the assessments were realized at the participating schools and performed by trained researchers. In addition, all adolescents received verbal instructions and an attempt to adapt and carry out the tests. Only the CRF assessment was performed once, as this is a maximum effort test.

### Anthropometry

Body mass was determined by using a portable digital reading scale (Seca, model 813, 0.1 kg precision). At the same time, height and trunk height were measured with a portable stadiometer (Harpenden, 0.1 kg precision), all according to Gordon et al. [23]. Skinfold thickness (tricipital and subscapular) was determined by a scientific adipometer (Lange®, Cambridge Scientific Instruments, Cambridge, MD), according to established methods [24].

For technical error assessment, 20% of the total sample was used. Adolescents had their anthropometric data collected two times, with one-week interval between measurements. The measurement error for height and skinfold thickness was low, with a high intraclass correlation coefficient (ICC > 0.965) in all determinations. The absolute technical error was 0.4 cm and 0.57 cm for height and trunk-head height, respectively, and 1.3 mm and 0.96 mm for the tricipital and subscapular skinfold, respectively.

### Somatic maturity

PHV was used to estimate the participants’ somatic maturity. The maturity offset or distance in years from PHV was estimated by the equation proposed by Mirwald et al. [25], using anthropometric data (height, sitting height and leg length), chronological age (CA) and body mass. The differential (ΔPHV) was calculated by the difference in years between the baseline and follow-up. In addition, the Mirwald’s equation was validated in a South American sample [26].

### Cardiorespiratory fitness (CRF)

The 20-m shuttle run test (SR-20 m) was employed to assess CRF, according to Léger et al. [27] instructions. Every subject had only one try to do the test, and all adolescents were verbally encouraged to perform their best during the test. SR-20 m is an intermittent 20 m run with an initial speed of 8.5 km/h that undergoes an increase of 0.5 km/h per minute. The test was interrupted if the participant did not achieve the 20 m distance on 3 consecutive times. Maximum oxygen consumption (VO_2max_) was calculated from the quadratic equation proposed by Mahar et al. [28]. The differential (ΔVO_2max_) was calculated by VO_2max_ follow-up—VO_2max_ baseline. The SR-20 m and Mahar’s equation was previously validated and recommended to use as a field test to assess CRF [29].

### Cognitive control tests

#### Corsi blocks

The Corsi block-tapping test (CB) was performed to assess the visuo-spatial short-term working memory. All participants were asked to perform the task once before the test to adapt and learn. CB test consists of memorizing cubes that flash on a computer screen. Initially, two cubes flash amid nine cubes arranged on the screen, at an increasing speed. The participants must reproduce the sequence in the same order, and increasingly longer strings are generated until their performance limit is reached. The test was interrupted whenever the participant did not memorize the sequence, marking two wrong answers at the same test level. The test period (Block span) (length of the last correct sequence) and the total score (Block span*correct answers in last stage) were adopted as performance indicators [30, 31].

#### Stroop test

The Stroop colour-naming task was performed to evaluate fast and accurate responding to colour stimuli. The test refers to the time length for an individual to name a colour when the stimulus word matches the stimulus’ colour (congruent condition) and when it does not (incongruent condition). Four different colours were used. The subjects performed 100 random stimulus-responses of the congruent and incongruous tasks, in which the inhibitory control (% of incongruent responses -% of congruent responses), the stimuli reaction time and the score were examined. All individuals performed the task once before the test for adaptation purposes [32, 33]. The Stroop test has been previously validated for a Brazilian sample [34].

### Sociodemographic variables

A questionnaire proposed by the Brazilian Association of Research Companies [35] was used to identify the students' socioeconomic level. Participants were classified into six economic groups (from 1 to 6 in ascending order of purchasing power). The mothers' education degree was also assessed according to six possible levels (1—unfinished elementary school; 2—complete elementary school; 3—unfinished high school; 4—complete high school; 5—unfinished higher education; and 6 – complete higher education).

### Statistical analysis

Data normality was assessed by asymmetry and kurtosis. The variables that did not exhibit a normal distribution were converted to log_10_. Comparisons between baseline and follow-up were computed using the T-test for paired samples, and the results were expressed as mean ± standard deviation. For the drop-out analysis, students that remained in both data collections were compared with drop-out ones. A multiple linear regression analysis was performed to identify the relationship between cardiorespiratory fitness and cognitive control variables (dependent variable), using confounding variables (i.e. sex, socioeconomic level, mother’s education, and body composition) [36]. The regression models included only the correlations that showed *P* < 0.10. The dependent variable was set as variables related to cognitive functions and independent variables related to cardiorespiratory fitness. For the regression analysis were realized the diagnosis of collinearity and the residual effects were also verified.

The significant regression models were tested for the mediations, assuming the PHV in follow-up as mediator for the analysis with the VO_2max_ in follow-up, and ΔPHV as mediator for the model with the ΔVO_2max._ The mediation analyses were performed according to the presumption proposed by Baron and Kenny [37]. Three hypotheses were assumed: (a) in the first equation, the independent variable must be a predictor of the mediating variable; (b) in the second equation, the mediating variable must be a predictor of the dependent variable, and controlled by the independent; and (c) the mediating variable must reduce the association between the independent and dependent variables. All analyses were performed using the SPSS V.26 software, at a 5% significance level.

## Results

The drop-out analysis did not show significant differences between students who remained both data collections (*n* = 134) with their peers (*n* = 239) for the main characteristic variables. Comparisons can be found in Supplementary File 1.

Table 1 shows the sample characterization in the baseline and the follow-up according to sex. Greater body mass, height and PHV were determined in the follow-up (all *p* < 0,05) in both sex. There was an increase in the sum of skinfolds (tricipital + subscapular) in girls (Δ = -0.9 mm; *p* < 0.05) and a decrease in boys (Δ = -0.9 mm; *p* < 0.05). Differences in CRF across sex were also found, with a 1.5 mL.kg^−1^.min^−1^ decrease in girls and a 2.7 mL.kg^−1^.min^−1^ increase in boys. Further details on the results of the cognitive function tests are shown in Table 1.

**Table 1:** Sample characterisation over three years at two data collection times: baseline and follow-up

	**Boys (65)**			**Girls (69)**			
	**Baseline**	**Follow-up**	**Δ**	**Baseline**	**Follow-up**		**Δ**
**Age (years)**	11.7 ± 0.6	14.9 ± 0.7*	3.2	11.6 ± 0.5	14.9 ± 0.5*		3.2
**Body weight (kg)**	46.1 ± 11.9	63.0 ± 13.9*	16.9	49.1 ± 13.3	61.1 ± 14.8*		12.0
**Height (cm)**	150.1 ± 7.0	170.8 ± 6.8*	20.7	153.4 ± 7.4	163.0 ± 7.1*		9.5
**∑ skinfold (mm)**	31.7 ± 16.6	30.7 ± 16.0	-0.9	34.7 ± 14.2	43.8 ± 15.4*		9.1
**VO**_**2max**_** (mL.kg**^**−1**^**.min**^**−1**^**)**	46.5 ± 6.3	49.3 ± 5.5*	2.7	39.4 ± 5.3	37.9 ± 5.9*		-1.5
**PHV (years)**	-1.84 ± 0.6	1.0 ± 0.9*	2.8	-0.02 ± 0.6	2.3 ± 0.5*		2.2
**Socioeconomic group**	-	4.9 ± 0.9		-	4.5 ± 1.0		
**Mothers’ education degree**	-	3.4 ± 1.6		-	3.3 ± 1.7		
**Stroop Test**							
Congruent Accuracy (%)	-	97.9 ± 3.0		-	99.6 ± 0.96		
Incongruent Accuracy (%)	-	96.8 ± 7.0		-	95.5 ± 10.9		
Congruent Reaction Time (s)	-	0.85 ± 0.1		-	0.93 ± 0.1		
Incongruent Reaction Time (s)	-	0.9 ± 0.2		-	1.06 ± 0.2		
Inhibitory Control (%)	-	3.0 ± 6.6		-	4.3 ± 10.7		
**Corsi Blocks Test**							
Block Span	-	6.0 ± 1.0		-	5.6 ± 1.1		
Total Score	-	54.7 ± 19.8		-	48.1 ± 19.5		

Results were expressed as mean ± standard deviation. Δ represents the mean difference between follow-up and baseline

Socioeconomic group, mothers’ education degree, Stroop test and Corsi Blocks test results were collected in the follow-up only

*PHV* Peak Height Velocity

^*^ Significant difference between baseline and follow-up at *p* < 0.05

The multiple linear regressions shown in Table 2 indicate an inverse significant relationship between CRF in the follow-up with congruent and incongruent reaction time, according to the values of the direct effects. VO_2max_ in the follow-up represented about 16% to 13% of the reaction time. Meanwhile, the variation in VO_2max_ values between baseline and follow-up pointed out to a significant impact of the congruent reaction time (β = -0.006; CI = -0.009 – -0.002), with an explanatory power of 18%, and a significant impact in incongruent reaction time (β = -0.006; CI = -0.011 – -0.001). Also, a tendency was found for the transversal relationship between CRF and the inhibitory control (β = -0.006; *p* = 0.06*).*

**Table 2:** Linear regression models between maximum oxygen level and cognitive functions (*n* = 134)

Log Congruent Reaction Time (s)	β ^(CI 95%)^	R^2^	p
Δ VO_2max_	^**−0.006 (−0.009 – −0.002)**^	**0.18**	**0.001**
VO_2max_ follow-up	^**−0.004 (−0.007 – −0.002)**^	**0.16**	**0.001**
**Log Incongruent Reaction Time (s)**	**β **^**(CI 95%)**^	**R**^**2**^	**p**
Δ VO_2max_	^**−0.006 (−0.011 – −0.001)**^	**0.15**	**0.012**
VO_2max_ follow-up	^**−0.005 (−0.008 – −0.001)**^	**0.13**	**0.004**
**Inhibitory Control (%)**	**β **^**(CI 95%)**^	**R**^**2**^	**p**
Δ VO_2max_	^−0.294 (−0.694 – 0.107)^	0.08	0.149
VO_2max_ follow-up	^−0.270 (−0.559 – 0.018)^	0.06	0.066
**Block Span**	**β **^**(CI 95%)**^	**R**^**2**^	**p**
Δ VO_2max_	^0.037 (−0.010 – 0.083)^	0.08	0.123
VO_2max_ follow-up	^0.020 (−0.012 – 0.052)^	0.06	0.209
**Total Score**	**β **^**(CI 95%)**^	**R**^**2**^	
Δ VO_2max_	^0.632 (−0.222 – 1.486)^	0.05	0.146
VO_2max_ follow-up	^0.413 (−0.166 – 0.992)^	0.05	0.161

The analyses were controlled by sex, mothers’ education and socioeconomic level. In the Δ VO_2max_ computations, VO_2max_ was added as a control in the baseline; CI 95%: 95% Confidence Interval; R^2^: model explanation coefficient. Variables in bold are significant at *p* < 0.05. Dependent variable: in bold writing

**Fig. 2:**
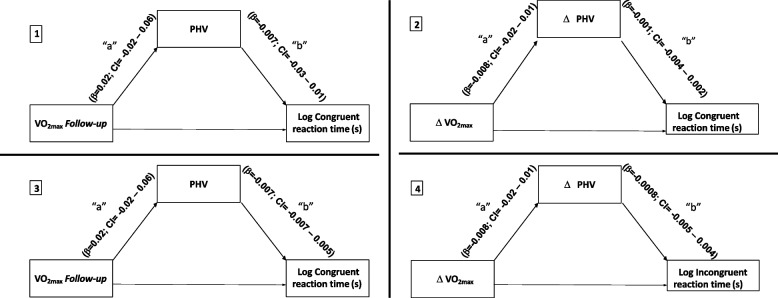
Mediation models of PHV and the relationship between the maximal oxygen consumption and functions of cognitive control. CI: 95% confidence interval

Figure 2 shows the PHV mediation model for the cognitive control variables (congruent and incongruent reaction time) that significantly correlate with CRF. This direct relationship is presented in Table 2. The pathway “a” was not significant, which represents the relationship between PHV and CRF (β = 0.02; CI = -0.02 – 0.06) or ΔPHV and ΔCRF (β = -0.01; CI = -0.02 – 0.01). Also, pathway “b,” which means the relationship of the mediator variable with congruent/incongruent reaction time, controlled by CRF, did not show significance. In this case, the presumption for mediation is violated.

## Discussion

The purpose of this study was to test the mediating role of somatic maturity (through PHV) on the associations between CRF and cognitive control functions. We found significant associations between CRF and both reaction time and inhibitory control. Higher VO_2max_ levels resulted in shorter reaction time, indicating a beneficial effect of improving CRF over the years on cognitive control. However, PHV did not seem to mediate the association of CRF with cognitive control functions.

Associations between CRF and cognitive control functions demonstrate that the higher the VO_2max_, the shorter the reaction time in cognitive control tasks and the greater the inhibitory control. The increase in CRF was also significantly associated with reaction time, with an explanatory power of 18%. Another study reports a similar conclusion, in which the CRF of children (aged 8–10 years) has an explanatory power of 19% concerning reaction time in congruent responses and 20% in incongruent responses [19]. Bruijn et al. [10] found direct and significant associations between CRF and inhibitory control (β = 0.15). Such associations may be explained by higher levels of CRF, leading to morphological changes and brain activation, thereby resulting in improved synapses and thicker frontal cortex and basal nuclei [11, 17]. The consequent lower neural interference can promote greater efficiency of the cognitive system and speed up signal processing [11, 12, 17, 38].

The associations between the mediator variable (PHV/ΔPHV) and both CRF and cognitive control functions were not statistically significant, thereby indicating that somatic maturity did not affect the relationship between CRF and cognitive control. Such a result can be attributable to the large number of individuals past the PHV in the sample. However, the better theory explanation is that the body systems development and changes in body size do not necessarily occur in parallel with the development of the cognitive system and other systems. The general growth presented a sigmoid pattern, whereas the neural development has a logarithmic pattern [2]. With these distinct times, the somatic maturity (dependent on body size) seems not to follow the specific time of neural growth [2]. More significant effects are likely observed when associating sexual hormones with the cognitive control as such hormones are provided with receptors in the cytoplasm of neural cells, increasing synapses and enhancing stimulation of the frontal and prefrontal cortex, which are responsible for the cognitive control functions [14, 16].

The limitations of the study were that cognitive control functions and the potential confounders were assessed exclusively in the follow-up, and such analyses were not grouped by sex. In addition, it was identified that the Corsi block test was not validated for this sample. In addition, this study does not have the reliability of cognitive tests. Regarding PHV, it was observed that the sample of the present study in the Follow-up phase has predominance of adolescents who went through PHV. Despite that, the highlights of this research were the evaluation of the mediating role of somatic maturity on different cognitive control functions and the CRF longitudinal character. Further studies should assess the mediating role of other maturation processes on CRF and cognitive control functions.

## Conclusions

CRF was shown to be a possible predictor of cognitive functions in cross-sectional, also the increase of CRF presented associations with the cognitive functions, suggesting that the promotion of increased VO_2max_ levels results in better reaction times, regardless of the value range examined. However, changes in body growth (assessed in terms of somatic maturity) did not impact the associations of CRF with reaction time or with inhibitory control. Thus, future studies with adolescents should focus on the impacts of other maturation processes on CRF and cognitive control.

## Supplementary Information

** Additional file 1: Supplementary Table 1.** Drop-out analysis.

## Data Availability

The datasets used and/or analyzed during the current study are available from the corresponding author on reasonable request.

## Abbreviations

ΔPHV: Variation in Peak High Velocity
ΔVO_2max_: Variation in maximum oxygen uptake
CA: Chronological Age
CB: Corsi Block
CRF: Cardiorespiratory Fitness
ICC: Intraclass correlation coefficient
PHV: Peak Height Velocity
VO_2max_: Maximum oxygen uptake
