# Supplementary material for: Association between cardiorrespiratory fitness and cognitive control: is somatic maturity an important mediator?
Source: BMC Pediatr. 2022 Dec 6;22:699. doi: 10.1186/s12887-022-03777-2 (PMC9724316; doi:10.1186/s12887-022-03777-2)
Supplement: Supplementary file 1 — Additional file 1: Supplementary Table 1. Drop-out analysis. [file 12887_2022_3777_MOESM1_ESM.docx]

Supplementary Table 1 - Drop-out analysis

|  | **Girls** | |  | **Boys** | |  |
| --- | --- | --- | --- | --- | --- | --- |
|  | **Remain (n=69)** | **Drop-out (n=136)** | ***P*** | **Remain (n=65)** | **Drop-out (n=124)** | ***P*** |
| **Age (years)** | 11.0 (11.0 - 12.0) | 11.0 (11.0 – 12.0) | 0.51 | 11.0 (11.0 – 12.0) | 11.0 (11.0 – 12.0) | 0.20 |
| **Weight (kg)** | 46.4 (39.2 – 54.6) | 44.2 (37.9 – 53.1) | 0.27 | 43.5 (37.2 – 54.2) | 44.0 (35.2 – 54.0) | 0.68 |
| **Height (cm)** | 151.7 (148.6 – 158.8) | 152.7 (146.3 – 158.1) | 0.50 | 150.8 (145.4 – 154.9) | 149.9 (145.0 – 156.2) | 0.90 |
| **Waist Circumference (cm)** | 66.0 (60.2 – 74.3) | 64.2 (59.3 – 71.0) | 0.41 | 68.5 (63.0 – 77.6) | 67.0 (60.5 – 75.5) | 0.12 |
| **Sum of Skinfolds (mm)** | 33.0 (22.0 – 44.0) | 28.0 (20.0 – 40.0) | 0.12 | 26.5 (17.0 – 44.7) | 23.0 (16.0 – 40.0) | 0.15 |
| **Peak high Velocity (years)** | -0.1 (-0.3 – 0.3) | -0.01 (-0.5 – 0.5) | 0.96 | -1.9 (-2.2 - -1.4) | -1.9 (-2.3 - -1.3) | 0.95 |
| **Shuttle run test (minutes)** | 3.1 (2.3 – 4.1) | 3.1 (2.3 – 4.4) | 0.88 | 4.4 (2.8 – 5.6) | 4.6 (3.1 – 6.2) | 0.24 |

Note: The values are expressed in median and interquartile range (25 – 75).
